# Case Report: New insights about clinical manifestations of patients with *GCK* genetic variants

**DOI:** 10.3389/fendo.2025.1549279

**Published:** 2025-04-15

**Authors:** Ritiele Bastos de Souza, Gabriella de Medeiros Abreu, Marília Chaves Bernardo, Roberta Magalhães Tarantino, Melanie Rodacki, Lenita Zajdenverg, Amanda Ferreira de Andrade, Deborah Snaider Nicolay, Ana Carolina Proença da Fonseca, Kaio Cezar Rodrigues Salum, Renata Szundy Berardo, Jorge Luiz Luescher, Verônica Marques Zembrzuski, Pedro Hernan Cabello, Mario Campos Junior

**Affiliations:** ^1^ Laboratory of Human Genetics, Oswaldo Cruz Institute, Oswaldo Cruz Foundation, Rio de Janeiro, Brazil; ^2^ Josué de Castro Nutrition Institute, Federal University of Rio de Janeiro, Rio de Janeiro, Brazil; ^3^ Diabetes and Nutrology Section, Internal Medicine Department, Federal University of Rio de Janeiro, Rio de Janeiro, Brazil; ^4^ Laboratory of Immunopharmacology, Oswaldo Cruz Institute, Oswaldo Cruz Foundation, Rio de Janeiro, Brazil; ^5^ Genetics Laboratory, Grande Rio University/AFYA, Rio de Janeiro, Brazil; ^6^ Postgraduate Program in Translational Biomedicine, Grande Rio University/AFYA, Rio de Janeiro, Brazil; ^7^ Clementino Fraga Filho University Hospital, Federal University of Rio de Janeiro, Rio de Janeiro, Brazil; ^8^ Pediatric Endocrinology Section, Federal Hospital of State Servants, Rio de Janeiro, Brazil; ^9^ Martagão Gesteira Child Care and Pediatrics Institute, Federal University of Rio de Janeiro, Rio de Janeiro, Brazil

**Keywords:** monogenic diabetes, GCK-MODY, glucokinase, screening, atypical symptoms

## Abstract

GCK-MODY is a genetic condition characterized by alterations in the *GCK* gene, which can include several types of inactivating genetic variants - ranging from missense and nonsense variants, and splice site variants, to small and large deletions and insertions in the gene. This disorder primarily affects glucose homeostasis and usually presents in heterozygous individuals. Although GCK-MODY is a well-studied condition, some variant carriers may manifest symptoms that deviate from the typical disease phenotype. Our study identified two Brazilian patients with GCK-MODY carrying novel frameshift variants, one of whom presented atypical manifestations of the disease. The patient is a 14-year-old male harboring a variant c.398del; p.(Phe133SerfsTer7) in the *GCK* gene. He presented with the typical clinical features of GCK-MODY, including mild and stable fasting hyperglycemia, however, he also presented a history of polyuria and polydipsia, which are unusual symptoms of the disease. These symptoms could be associated with the more severe impact of a frameshift variant. However, we did not observe the same unusual phenotype in our second patient, who is a 15-year-old normal-weight female. At the age of 8, she was diagnosed with diabetes mellitus. The patient with the p.(Val335ArgfsTer124) variant presented with mild, stable hyperglycemia, a characteristic feature of the disease. In this study, we present two cases of novel frameshift variants in *GCK* and review other reports in the literature that have shown patients with atypical manifestations of the disease and highlight the importance of a comprehensive characterization of the phenotypic spectrum caused by GCK-MODY variants.

## 1 Introduction

Maturity-onset diabetes of the young (MODY) is a type of diabetes mellitus (DM) that has a genetic etiology and is considered the most prevalent form of monogenic diabetes mellitus (1). MODY manifests as early-onset hyperglycemia, typically occurring before the age of 25 years. Additionally, patients with MODY often exhibit a familial recurrence of the disease across two or more successive generations, alterations in pancreatic β-cell function, and a lack of β-cell autoimmunity (2). MODY is mainly inherited in an autosomal dominant pattern and results from variants found in one of several genes including *HNF1A*, *GCK*, *HNF4A*, *HNF1B*, *PDX1*, *NEUROD1*, *KLF11*, *CEL*, *PAX4, INS*, *BLK, ABCC8*, *KCNJ11*, *APPL1*. This disease is heterogeneous and one of its most common forms is caused by variants in the *GCK* gene, leading to a clinical condition known as GCK-MODY (3–6).

GCK-MODY is a genetic disorder that can be caused by different types of inactivating genetic variants such as missense and nonsense variations, splicing variants, small and large deletions, and insertions. This disorder primarily affects glucose homeostasis and is usually observed in heterozygosity (7–9). The *GCK* gene, responsible for this disorder, is situated on chromosome 7p13 (OMIM138079) and comprises a total of 10 exons that encode the enzyme glucokinase/hexokinase IV. This enzyme consists of 465 amino acids and is expressed in pancreatic β cells, hepatocytes, brain, and endocrine cells of the gut (10). Glucokinase plays a vital role in the glycolysis pathway by converting glucose to glucose-6-phosphate (G6P), which is dependent on adenosine triphosphate (ATP). This reaction primarily occurs in pancreatic β cells due to the increase in glucose concentration and enzyme activation and is responsible for promoting an increase in the ATP/ADP ratio, which results in the closure of potassium channels (K+ATP), depolarization of the membrane, opening of calcium channels (Ca2+) and the release of insulin granules by beta cells (11, 12).

GCK-MODY is a clinical condition with distinct features concerning typical DM. Patients with GCK-MODY present stable fasting hyperglycemia of 99–153mg/dL (5,5–8,5 mmol/L) (12), that is non-progressive, and their HbA1c (Glycated hemoglobin) levels range from 5,6-7,3% (38–56 mmol/mol) before the age of 40 years, and 5,9-7,6% (41–60 mmol/mol) after the age of 40 years (5, 13). Furthermore, patients with GCK-MODY demonstrate a low chance of developing microvascular or macrovascular complications related to other forms of diabetes, despite mild hyperglycemia since birth (14, 15).

The therapeutic management of GCK-MODY requires a different approach compared to other forms of diabetes. In most cases, these patients generally do not need antihyperglycemic pharmacological therapy (16). However, a concern for patients with GCK-MODY is that, due to showing a mild form of asymptomatic hyperglycemia, it often goes underdiagnosed and is usually detected accidentally during routine medical tests and often misdiagnosed as type 1 or type 2 diabetes mellitus (17, 18).

Although the clinical phenotype of GCK-MODY is well established and known, recent studies suggest that some patients with GCK-MODY may present slightly more severe or atypical symptoms of the disease. It is suggested that these differentiated clinical manifestations may be due to the type of variant identified in the *GCK* gene (19) or possibly gender differences could impact phenotype expression; or other factors such as penetrance variability, environmental or lifestyle influences, or simple chance (20). In addition, it was recently seen that increased weight/obesity could be a contributing factor to the increased HbA1c levels of GCK-MODY patients, posing a risk for diabetic complications (21).

Recent studies have indicated that phenotypic manifestations such as polyuria and polydipsia, typically observed in patients with HNF1A-MODY (18), have also been observed in patients with GCK-MODY in recent years (20, 22–24). This highlights the importance of identifying the factors associated with the potentially more severe clinical manifestations of GCK-MODY. A comprehensive understanding of these cases could facilitate the avoidance of inappropriate therapeutic interventions for this condition.

The purpose of this study was to describe the clinical features of two patients from Rio de Janeiro with GCK-MODY that carried distinct novel frameshift variants (c.398del; p.(Phe133SerfsTer7) and c.1002dup; p.(Val335ArgfsTer124)), one of which presented atypical features of the disease. Additionally, a comprehensive literature review was conducted to investigate the variants observed in GCK-MODY patients manifesting atypical symptoms.

## 2 Cases presentation

### 2.1 Patient 1

**Figure 1:**
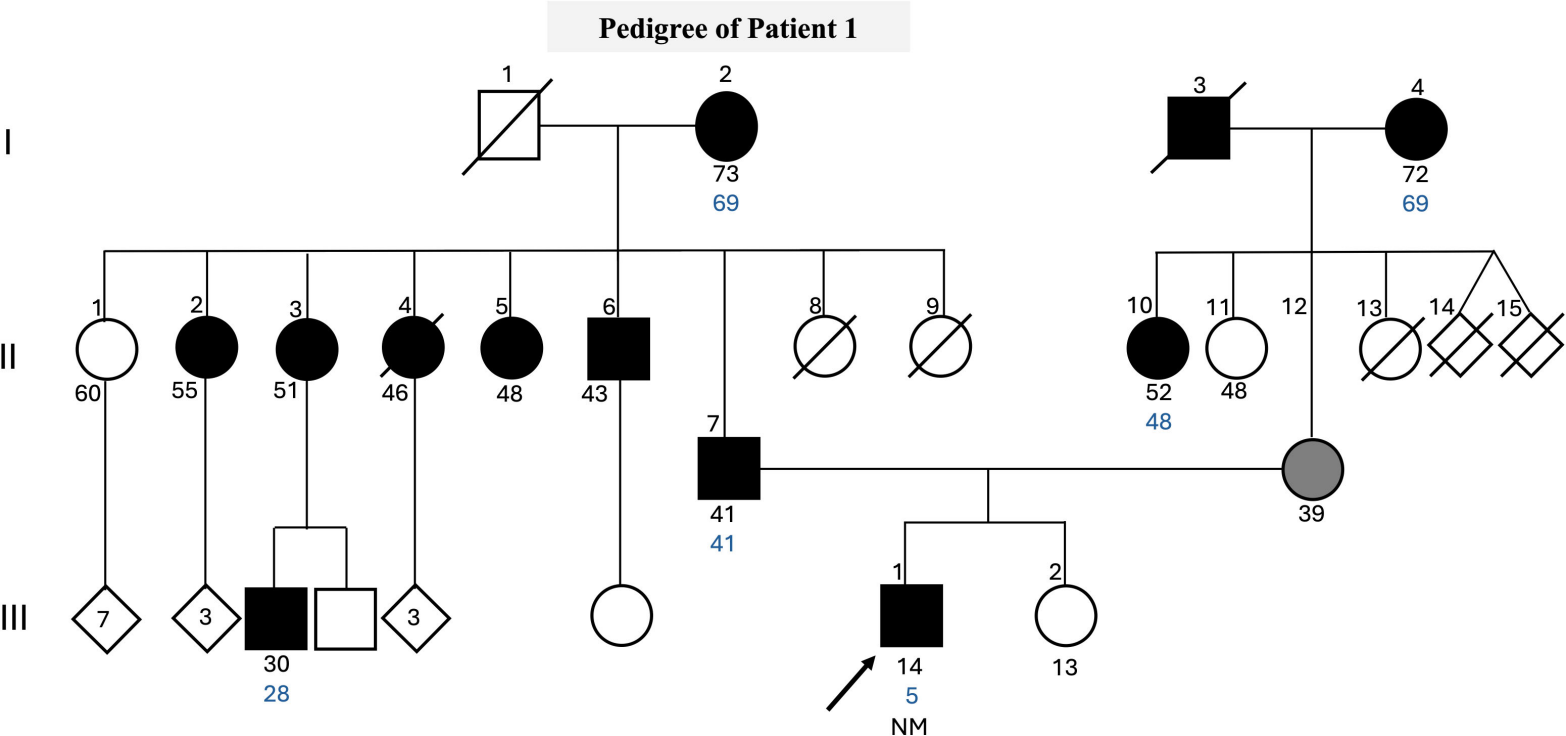
Pedigree clinical characteristics of the family. The symbols filled with black represent individuals with hyperglycemia, while the gray-filled symbols represent individuals with gestational diabetes, and the empty symbols represent non-hyperglycemic individuals. The age in years of the individuals is shown below the symbols, and the age of diagnosis is in blue. Genotypes of *GCK* c.398del are expressed by normal allele (N) and mutated allele (M). An arrow indicates the index case.

We performed a genetic screening analysis of the coding sequence of the *GCK* gene in a 14-year-old male participant. The patient was diagnosed with DM at the age of 5 years old with fasting blood glucose of 121 mg/dL, HbA1c of 7.8%, and a history of polyuria, polydipsia, and absence of diabetic ketoacidosis. Insulin therapy was initiated at the time of diagnosis with NPH (Neutral Protamine Hagedorn) and regular insulin. At the age of 12, he tested negative for Glutamic acid decarboxylase (GAD) antibody (1.8 UI/mL) and had a C-peptide level of 1.7 ng/mL. Throughout the treatment course, the patient maintained effective glycemic control with HbA1c averaging between 6.5% and 8.5%. He is currently prescribed a daily regimen of NPH insulin at a dosage of 0.31 units per kilogram. In recent months, without the need for regular insulin application. If his blood glucose levels are equal to or exceed 200 mg/dL, he supplements his dosage with regular insulin. The patient has reported a family history of type 2 DM (**Figure 1**). This patient has also been tested for variants in *HNF1A*, *HNF4A*, *HNF1B*, *PDX1*, *PAX4*, *NEUROD1*, *KLF11*, *INS*, *MT-TL1*, and *KCNJ11.*

### 2.2 Patient 2

**Figure 2:**
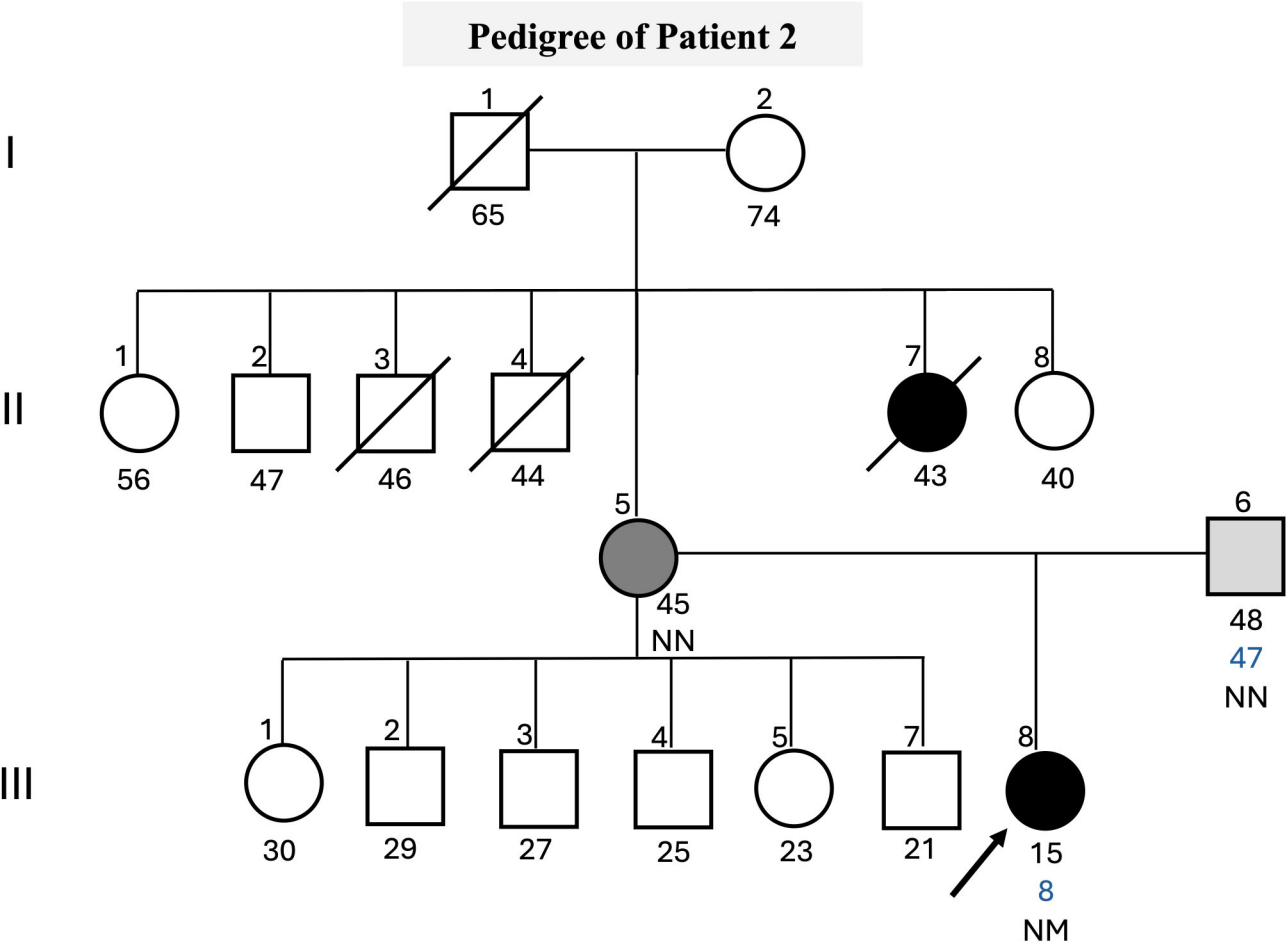
Pedigree, clinical characteristics of the family. The symbols filled with black represent individuals with hyperglycemia, the gray-filled symbols represent individuals with gestational diabetes, the light gray symbols represent individuals with prediabetes, and the empty symbols represent non-hyperglycemic individuals. The age in years of the individuals is shown below the symbols, and the age of diagnosis is in blue. Genotypes of *GCK* c.1002dup are expressed by normal allele (N) and mutated allele (M); An arrow indicates the index case. The index case carries a c.1002dup; p.(Val335ArgfsTer124) variant, which is absent in both parents (NN genotype), suggesting a *de novo* mutation.

The proband 2 is a 15-year-old normal-weight female, who underwent genetic screening of the *GCK* gene. At the age of 8, she was diagnosed with diabetes mellitus after experiencing malaise, tachycardia, and fainting twice. During the investigation of her case, altered blood glucose levels were detected by the oral glucose tolerance test (OGTT) (139 mg/dL). Tests were negative for anti-GAD and anti-IA2 autoantibodies. At the age of 10 years old, she was hospitalized due to an infection with the Chikungunya virus, resulting in elevated blood glucose levels, that reached approximately 400 mg/dL. However, this was an exceptional occurrence of hyperglycemia during the clinical evolution of the case. During routine consultations, when the patient was between 12 and 15 years old, the patient’s C-peptide, fasting and postprandial blood glucose, and HbA1c levels were monitored on different dates, and values close to compliance were observed. Two years after diagnosis in February 2019, the C-peptide measurement was 6.0 ng/mL. Variations in blood glucose levels were monitored, fasting blood glucose levels ranged from 106 to 122 mg/dL, and postprandial blood glucose levels ranged from 136 mg/dL to 169 mg/dL. The patient’s HbA1c levels ranged from 5.9% to 6.9%. In May 2024, the average glucose level was 109 mg/dL on continuous glucose monitoring (CGM) over a 14-day period. The CGM data showed that glucose levels were within the target range of 70 to 180 mg/dL for 99% of the time, while 1% of the time was spent with values between 181 and 250 mg/dL. The patient has reported a family history of gestational diabetes and prediabetes (**Figure 2**).

## 3 Molecular analysis

Genomic DNA was extracted from leukocytes using a commercial kit by QIAamp DNA Blood Mini Kit (Qiagen, Hilden, Germany). The quality and concentration of DNA was evaluated using the NanoDrop^®^ spectrophotometer. The amplification of the coding regions of the *GCK* gene (NM_000162.5), and flanking intronic sequences was carried out in the patient’s sample using pairs of specific primers for each fragment of gene (**Supplementary Table S1**). The PCR products were purified using the ExoSAP-IT^®^ Reagent from Applied Biosystems (Vilnius, Lithuania). The products were screened by automatic Sanger sequencing using the Big Dye Terminator Kit v3.1 from Applied Biosystems (Austin, TX, USA). The sequencing was performed on an ABI 3730 automated sequencer from Applied Biosystems.

**Figure 3:**
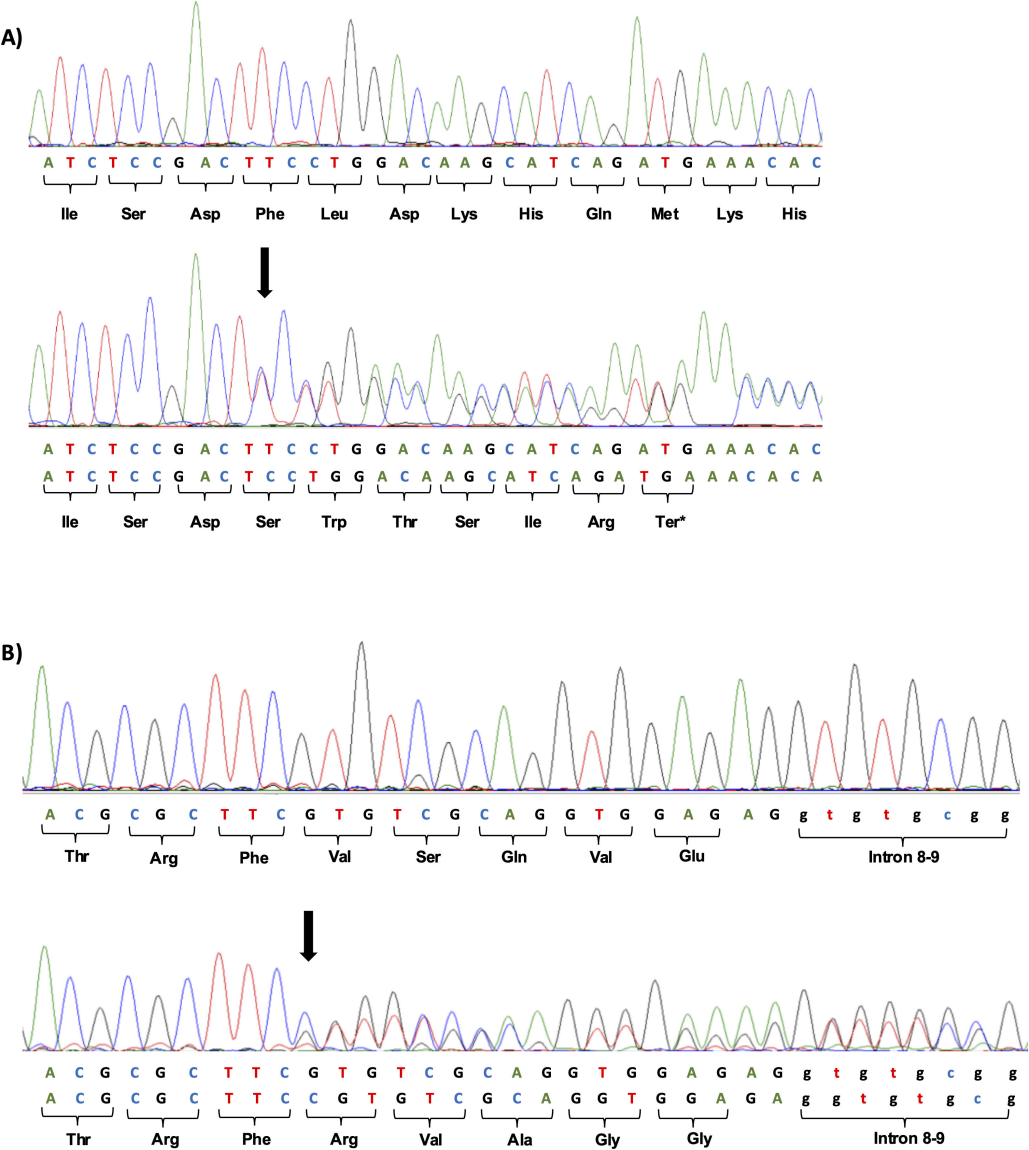
**(A)** Heterozygous frameshift variant c.398del; p.(Phe133SerfsTer7) identified in exon 4 of the *GCK* gene by Sanger sequencing. The wild sequence of part of exon 4 and altered sequence, with deletion of a T at position 398 of c.DNA is shown below. The location of the deletion is indicated by the black arrow. **(B)** Heterozygous frameshift variant c.1002dup; p.(Val335ArgfsTer124) identified in exon 8 of the *GCK* gene by Sanger sequencing. The wild sequence of part of exon 8 and altered sequence, with duplication of a C at position 1002 of c.DNA is shown below. The location of the duplication is indicated by the black arrow.

Two heterozygous frameshift variants were identified in two patients clinically suspected of MODY. The c.398del; p.(Phe133SerfsTer7) variant located in exon 4 (**Figure 3A**), was observed in patient 1. The second variant, c.1002dup; p.(Val335ArgfsTer124), located in exon 8 (**Figure 3B**), was observed in patient 2.

The two variants c.398del; p.(Phe133SerfsTer7) and c.1002dup; p.(Val335ArgfsTer124) were not found in the literature and are not present in any major genetic variant investigation databases such as gnomAD (exomes and genomes) (https://gnomad.broadinstitute.org/), dbSNP (https://www.ncbi.nlm.nih.gov/), ClinVar (https://www.ncbi.nlm.nih.gov/), HGMD (http://www.hgmd.cf.ac.uk/ac), and in the ABraOM (https://abraom.ib.usp.br/search.php), a Brazilian repository. The variants were classified as likely pathogenic based on ACMG automated criteria (PVS1, PM2) using the Franklin platform (https://franklin.genoox.com - Franklin by Genoox).

## 4 Discussion

In this study, we investigated two patients suspected of having MODY. These patients exhibit clinical characteristics consistent with the disease, including an onset of diabetes at or before 35 years of age and a body mass index (BMI) of less than 30 kg/m² or at the 95th percentile at the time of diagnosis. Both patients tested negative for β-cell autoantibodies, such as anti-glutamic acid decarboxylase (anti-GAD) and/or anti-IA-2. They do not have a history of diabetic ketoacidosis, show no clinical signs of insulin resistance, and no known secondary causes of their diabetes were observed.

The genetic screening revealed both patients had a frameshift variant in the *GCK* gene. The variants could result in the formation of truncated proteins, leading to premature termination of glucokinase protein synthesis. The c.398del; p.(Phe133SerfsTer7) variant caused a frameshift in the sequence starting at amino acid residue 133 and causing a premature termination seven amino acids after the deletion representing a loss os 71.4% of the reference sequence. The altered region starts in the small domain of the glucokinase protein. In contrast, the c.1002dup; p.(Val335ArgfsTer124) variant affects the coding of the large domain of the protein. This variation leads to a frameshift starting at amino acid residue 335, and ending in a premature stop 124 amino acids residues downstream, causing the loss of 28.0% of the reference sequence.

More than 900 variants in the *GCK* gene have been reported and described along this gene, leading to different phenotypes (9). The phenotype GCK-MODY is well-known for developing mild and non-progressive diabetes in most cases. However, some patients with *GCK* variants were reported with some variations in their clinical expression. Some Other studies have also observed varying clinical profiles depending on the variant identified in the *GCK* gene, demonstrating differences in fasting blood glucose levels, glucokinase activity, and glucose affinity (25, 26). These differences can be seen more clearly in some rare cases of individuals with homozygotic variants. Some variants in homozygosity can cause permanent neonatal diabetes while others a typical GCK-MODY phenotype (8, 27, 28).

Our patient observed with the c.398del; p.(Phe133SerfsTer7) variant showed typical clinical characteristics of GCK-MODY, which included mild and stable fasting hyperglycemia, and HbA1c within the expected range. However, he reported a history of polyuria and polydipsia, which are uncommon symptoms for GCK-MODY. Interestingly, Ping Xiao and collaborators reported a frameshift variant also in exon 4 (c.367-374dupTTCGACTA, p.(Ile126fs)) in an 11-year-old male patient with diabetes, who had been experiencing symptoms of polyuria and polydipsia for the past seven years, in addition to weight loss. The patient had a family history of hyperglycemia, and the same frameshift variant was detected in his sister, father, and grandmother (24).

Similarly, another study reported a large heterozygous deletion of the entire exon 1 (4763pb), covering the promoter region of the *GCK* gene in two unrelated male patients of French-Canadian descent with a clinical history of MODY. Both patients experienced osmotic symptoms such as polyuria and polydipsia. In addition, one of the probands also had fasting blood glucose levels above the expected range, up to 162 mg/dL. The authors suggested that large deletions in this gene could result in a more severe phenotype than those caused by other types of variants identified in GCK-MODY patients. The probands inherited the variant from their mothers, however, their mothers exhibited milder characteristics of DM, similar to the sister of proband 1, who presented the mild and more common phenotype of the GCK-MODY. Another hypothesis suggested by the authors is that this phenotypic difference may be linked to sex, variable penetrance, or environment (20).

Furthermore, another large deletion involving the final three exons of the *GCK* gene was described. The patient, a 31-year-old Caucasian female, was diagnosed with diabetes at the age of 21 years. Her symptoms started at the age of 19 with occurrences of fever and myalgias. At the age of 20, it was decided to introduce insulin therapy, using a basal-bolus scheme after experiencing glycemic decompensation with symptoms of polyuria, polydipsia, and unexplained weight loss during steroid therapy. The patient presented inflammatory and autoimmune diseases but did not have specific autoimmunity for type 1 diabetes. In addition, the patient reported that both his mother and grandmother had the onset of diabetes at the ages of 31 and 30, respectively (22).

Just as frameshift variants and large deletions/insertions can lead to the formation of premature or inactive proteins, nonsense variants or those affecting splicing regions can also have great impact in the protein. Some nonsense variants associated with GCK-MODY have been described in the literature. However, it appears that these patients have not exhibited any atypical or unusual characteristics of the disease. Only the classic symptoms have been observed in these cases (24, 29–31). The same scenario was not seen for changes affecting splicing regions. In 2018, Cho e colleagues identified a deletion of 79 nucleotides in the intron 9–exon 10 of the *GCK* gene that is predicted to disrupt the pre-mRNA splicing of *GCK*. It was found in a patient who had MODY phenotypes. The patient was diagnosed at nine years of age due to glycosuria detected in the school check-up program. This manifestation is rare in GCK-MODY patients. The patient’s pediatric physician prescribed insulin or metformin for several years but failed to achieve the target level of below 100 mg/dL. At the age of 17, the patient stopped clinical monitoring and taking medication. At the age of 21, even without treatment, medical tests showed a fasting blood glucose of 133 mg/dL and HbA1c of 6.4% (32).

Although the GCK-MODY cases mentioned with atypical symptoms for the disease appear to carry genetic variants that result in the premature and incomplete formation of the protein, it has already been seen that some missense variants can also be associated with these atypical features. Missense variants can have a varying impact on the protein, generating changes in the secondary structure, compromising stability, or causing the elimination of essential catalytic domains of the protein (23, 31).

One recent case was observed in Peru: a male patient carrying a previously known missense variant c.629C>T; p.(Thr210Met) in the *GCK* gene. He was diagnosed with DM at the age of 13 years and initiated treatment with metformin. He had no family history of first-degree with DM. Over a period of two years with sporadic health check-ups, the patient reported a loss of 2 kg in weight, persistent fatigue, and mild increases in thirst and urine output, without any evidence of insulin resistance characteristics, such as acanthosis nigricans or obesity. Initially diagnosed with type 2 diabetes, his condition was later reclassified as type 1 diabetes. He started using low-dose insulin for more than six months, but the investigation and clinical findings did not align with expectations for the most common types of DM. So, additional genetic testing led to the identification of a *de novo* heterozygous pathogenic variant in the *GCK* gene (23). In addition, in the same year, the study by Alvelos et al., 2020 (31) demonstrated the alteration c.1099G>A; p.(Val367Met) in a 15-year-old female patient, diagnosed at 14 years of age presenting signs and symptoms of polyuria and polydipsia. She had a BMI of 25.5 kg/m², C-Peptide of 4.50 ng/mL, no positivity for pancreatic autoantibody, and a glycated hemoglobin level of 6.5% before starting treatment with oral hypoglycemic agents.

The studies mentioning atypical manifestations of GCK-MODY were summarized in **Table 1**. This report describes two frameshift alterations: p.(Phe133SerfsTer7) and p.(Val335ArgfsTer124) in the *GCK* gene. These variants were observed in patients of different genders and were associated with some phenotypic differences. The patient carrying the p.(Phe133SerfsTer7) alteration showed uncommon manifestations, such as osmotic signs. These findings are consistent with the literature on this subject. On the other hand, the patient with the p.(Val335ArgfsTer124) variant showed more classic symptoms of the disease.

**Table 1:** Compilation of atypical clinical characteristics in patients with GCK-MODY.

Alterations	Exon/ intron	Sex	Diagnosis (Age)	BMI, kg/m2 or weight percentile	Fasting Glucose mmol/L (Diagnosis)	Fasting Glucose mmol/L (last collected)	HbA1c % (Diagnosis)	HbA1c % (last collected)	Autoantibodies (Anti GAD)	Family history	Treatment	Atypical clinical features	Participants’ Nationality	References
**p.(Phe133SerfsTer7)**	4	M	5	< P85	6.72	7.16	7.8	6.9	Negative 1,8	Mother; father; maternal grandfather; paternal uncle; paternal aunt.	NPH insulin (0.31U/kg/d) and regular insulin if blood glucose ≥ 200.	Symptoms such as polyuria and polydipsia.	Brazilian	Novel
**p.(Val335ArgfsTer124)**	8	F	8	<P85	7.71 (OGTT)	6.77	n.a	6.9	Negative	Father	Diet	Not	Brazilian	Novel
Other studies														
p.(Ile126fs)	4	M	11	14.82	7.7	7.2 (OGTT)	8.4	6.7	Negative	Father; sister; grandmother.	Not	Symptoms such as polyuria and polydipsia.	Chinese	(24)
CNV - Deletion of the entire exon 1 (4763pb)	1	M	8.5	P95	7.0	6.0–9.0	n.a	6.4–6.9	Negative <1	Mother; sister.	Metformin	Symptoms such as polyuria and polydipsia.	French Canadian	(20)
CNV - Deletion of the entire exon 1 (4763pb)	1	M	8.9	P25	n.a	n.a	n.a	5.9 a 7.0	Negative <1	Mother	Diet	Symptoms such as polyuria and polydipsia.	French Canadian	(20)
Large deletion involving the final three exons of the *GCK*	8, 9 and 10	F	21	n.a	n.a	6.22	n.a	6.3	Negative	Mother; grandmother.	Metformin500, 1 tablet/day; Insulin Glargine 2 UI/day; Insulin aspart 2 UI before breakfast, 2 UI before lunch, and 2 UI before dinner.	Glycemic decompensation with polyuria, polydipsia, and unexplained weight loss.	Ukrainian	(22)
Deletion of 79 nucleotides in the intron 9–exon 10	intron 9 - exon 10	M	9	16,7	6.88	7.38	6.4	6.4	Negative	Mother; maternal grandfather.	Insulin and metformin.	Symptoms such as glycosuria.	Korean	(32)
p.(Thr210Met)	6	M	13	P10-25	n.a	7,32	6.8	6.3	Negative	Not	Metformin	Weight loss, fatigue, mild polydipsia, and polyuria after two years of irregular follow-up.	Peruvian	(23)
p.(Val367Met)	9	F	14	25.5	n.a	n.a	6.5	6.4	n.a	Mother; maternal grandmother.	Oral hypoglycemic agents	Polyuria and polydipsia.	Portuguese	(31)

n.a, Not available; F, Female; M, Male; BMI, Body Mass Index; NPH, Neutral Protamine Hagedorn.

The search for a correlation between genotype and phenotype in GCK-MODY patients has been a topic of interest. In 2017, Wędrychowicz and Colleagues reported in a study conducted on a cohort of 37 patients with GCK-MODY, that a range of phenotypic presentations may be noticed, from a completely asymptomatic form of the disease to typical symptoms of other types of diabetes. In their cohort, 4 patients (11%) presented with typical hyperglycemia symptoms such as polydipsia, polyuria, and fatigue. However, the study did not find a clear correlation between the patients’ genotypes and their clinical characteristics (33). In a study conducted by Gersin and colleagues, they developed a comprehensive map of missense and nonsense variants in the glucokinase enzyme, assessing their functional consequences, including those causing GCK-MODY. The researchers found that almost all variants in the enzyme region between residues ~150 and 200 considerably reduced protein activity compared to other areas of the enzyme. The study also highlighted the central role of this region in the conformational dynamics of glucokinase. It is important to note that this study does not include other types of *GCK* variants like the ones found in our research. However, it does offer valuable insights into the variants, their locations in the enzyme, and their potential functional consequences linked to the loss of enzyme activity and the differential phenotypic effects exerted by each variant (34).

Our findings, as well as those reported in the literature, suggest that the extent to which a variant impacts protein structure can result in similar osmotic symptoms (polyuria and polydipsia) across individuals. However, variant impact may not be the only factor associated with phenotypic fluctuations. We have also observed a patient with a frameshift variant in exon 8 that presented the expected symptoms of GCK-MODY. As other individual factors may also influence the observed clinical characteristics, it is a challenging task to establish a clear relationship between genotype and phenotype. Although we identified two alterations that do not exhibit the same osmotic symptoms across individuals, we observed some consistency when considering the different types of *GCK* variants reported above.

Our study has some limitation, such as the absence of a segregation analysis of the *GCK* variant due to the unavailability of family members for investigation. We also acknowledge the absence of functional studies to demonstrate the pathogenic effect of these variants on glucokinase function. Another limitation was that not all monogenic diabetes-related genes were tested in our patients.

## 5 Conclusion

To date, most cases of GCK-MODY are typically identified accidentally. Due to mild hyperglycemia, which is frequently detected during routine medical examinations or in the context of other clinical conditions (12). Nonetheless, atypical cases such as those reported here show the importance of screening for the *GCK* gene in patients suspected of having monogenic diabetes, even among individuals who do not exhibit the characteristic features associated with GCK-MODY. Furthermore, this investigation highlights the necessity for a more comprehensive characterization of these patients.

## Data Availability

The original contributions presented in the study are included in the article/supplementary material, further inquiries can be directed to the corresponding author.
