# Supplementary material for: Case Report: New insights about clinical manifestations of patients with GCK genetic variants
Source: Front Endocrinol (Lausanne). 2025 Apr 15;16:1549279. doi: 10.3389/fendo.2025.1549279 (PMC12037322; doi:10.3389/fendo.2025.1549279)
Supplement: Supplementary file 1 [file DataSheet1.pdf]

Supplementary Material

Supplementary Tables

Supplemental Table S1. Primer Sequences and PCR Conditions

Gene	Primers	Sequences 5' 3'	Ta °C	Amplicon bp
GCK	E1F	GAAGGACACTAAGCCCCACAG	60	505
	E1R	GGCACCCCTGGCAAGACC		
	E2F	GGGTCAGAAGACAGAAGGAGGC	62	415
	E2R	CTGTCTCGGGCTGGCTGTG		
	E3F	CCTTAGTCCCTTGTGCCTTCC	62	388
	E3R	CCGCTCTCCCCACCCCTG		
	E4F	CAGCAGAGCATTCAGCAGTATC	60	690
	E4R	GGGGCTACATTTGAAGGCAGAG		
	E5-6F	CTCCAGTATATGTTAGCAGC	60	504
	E5-6R	GATACCCCAAGACCACCCAGG		
	E7F	CACTGAAGCAACCCAGGTCT	60	596
	E7R	GATCACCTGTCGGAAGGAAA		
	E8F	GAGGGAAAGACGTGAACCAG	62	438
	E8R	AGGCCCTAGTTTCCCATCC		
	E9-10F	CTGTCGGAGCGACACTCA	62	700
	E9-10R	ATGGAGCCTGGGTGCTGT		

Ta: annealing temperature of the primers; bp: base pairs; F: Forward; R: Reverse.

<u>PCR conditions:</u>

- **Protocol** – 50 ng of genomic DNA, 1 unit of Platinum® Taq DNA Polymerase High Fidelity (InvitrogenTM, Carlsbad, CA, USA), 1X High Fidelity PCR Buffer (InvitrogenTM, Carlsbad, CA, USA), 2 mM $MgSO_4$ (InvitrogenTM, Carlsbad, CA, USA), 0.2 mM of each dNTP (InvitrogenTM, Carlsbad, CA, USA), and 0.4 µM of each primer.

<u>Cycling conditions:</u>

- **Protocol** - 94 °C for 2 min for initial denaturation, followed by 35 cycles of 94 °C for 30 s, primer-specific annealing temperature (°C) for 30 s, and 72 °C for 1 min; and a final extension step of 72 °C for 10 min. The reaction is maintained at 4 °C indefinitely.
